# The relationship between coronary artery calcification and bone metabolic markers in maintenance hemodialysis patients

**DOI:** 10.1186/s12882-023-03286-z

**Published:** 2023-08-15

**Authors:** Lin Xiong, Qi-qi Chen, Yong Cheng, Yong-shu Lan, Jian-bo Yang, Xiang-qiong Wen, Xin Xie, Ting Kang, Wei-hua Wu, Santao Ou

**Affiliations:** 1 Department of Nephrology, The Affiliated Hospital of Southwest Medical University, 25th Taiping Street, Luzhou, 646000 Sichuan China; 2 Sichuan Clinical Research Center for Nephropathy, Luzhou, Sichuan China; 3 Department of Radiology, The Affiliated Hospital of Southwest Medical University, Luzhou, Sichuan China; 4 Department of Nuclear Medicine, The Affiliated Hospital of Southwest Medical University, Luzhou, Sichuan China; 5 Nuclear Medicine and Molecular Imaging Key Laboratory of Sichuan Province, Luzhou, Sichuan, China; China; 6 Academician (Expert) Workstation of Sichuan Province, Luzhou, Sichuan China

**Keywords:** Maintenance hemodialysis, Vascular calcification, Bone metabolic markers, Relationship

## Abstract

**Background:**

To study the influencing factors for coronary artery calcification (CAC) in maintenance hemodialysis (MHD) patients and the relationship between CAC and bone metabolism markers and to attempt to find a reliable marker linking vascular calcification and bone metabolism in MHD patients.

**Methods:**

A total of 123 patients were enrolled. CAC was assessed by multislice spiral computed tomography (MSCT), and the CAC score (CACS) was evaluated using the Agaston method. Routine laboratory parameters, including triglycerides (TG), total cholesterol (TC), glucose (Glu), calcium (Ca), phosphorus (P), magnesium (Mg), etc., were measured. Serum markers of bone metabolism, such as alkaline phosphatase(ALP), calcitonin (CT), 25-hydroxy vitamin D [25-(OH)D], intact parathyroid hormone (iPTH), total type I procollagen amino-terminal peptide (tPINP), N-terminal mid-fragment of osteocalcin (N-MID OC), and β-type I collagen crosslinked carboxyl-terminal peptide (β-CTX), were also measured.

**Results:**

Among 123 MHD patients, 37 patients (30.08%) did not have CAC, and 86 patients (69.92%) had CAC, including 41 patients (47.67%) with mild calcification and 45 patients (52.33%) with moderate to severe calcification. Age, Body Mass Index(BMI), the prevalence of hypertension and diabetes mellitus, TC, Glu, P, and Ca×P in the calcification group were higher than those in the noncalcification group, whereas Mg, iPTH, tPINP, N-MID OC, and β-CTX were lower than those in the noncalcified group (*P* < 0.05). Compared with the mild calcification group (0<CACS<400), P and Ca×P levels were higher in the moderate to severe calcification group (CACS ≥ 400), and ALP, iPTH, N-MID OC, tPINP, and β-CTX concentrations were lower (*P* < 0.05). Correlation analysis showed that the CACS was positively correlated with TC, LDL-C, P, and Ca×P (*P* < 0.05) and negatively correlated with N-MID OC and β-CTX (*P* < 0.05). There was no significant correlation between the CACS and other parameters (*P* > 0.05). A logistic regression model was used to evaluate the influencing factors for CAC. The results showed that age, BMI, TC, Glu, P, and Ca×P were risk factors for CAC and its severity in MHD patients, whereas diabetes mellitus, Mg, and N-MID OC were protective factors for CAC in MHD patients. In addition, N-MID OC was a protective factor for the severity of CAC. After adjusting for the corresponding confounding factors, the results of the risk factors were consistent, and N-MID OC was still an independent protective factor for CAC and its severity.

**Conclusions:**

Elevated serum P and Ca×P were independent risk factors for CAC in MHD patients, and serum Mg may be an independent protective factor for CAC. CAC was closely related to abnormal bone metabolism and bone metabolic markers in MHD patients. Relatively low bone turnover can promote the occurrence and development of CAC. N-MID OC may be a reliable bone metabolic marker linking vascular calcification and bone metabolism in MHD patients.

## Introduction

Chronic kidney disease (CKD) is a growing health problem worldwide, affecting approximately 10%~16% of the adult population worldwide [1]. The prevalence of CKD in China has reached 10.8% [2], and the number of maintenance hemodialysis (MHD) patients is also gradually increasing. Cardiovascular disease is one of the most common complications in MHD patients and is closely associated with increased mortality. In addition, vascular calcification (VC) has been implicated as an independent risk factor for cardiovascular diseases [3]. The China Dialysis Calcification Study (CDCS) found that the incidence of CAC was the highest in MHD patients, reaching 72.4% [4].

Increasing evidence suggests that VC is an active process involving a phenotypic change of vascular smooth muscle cells (VSMCs) toward osteoblast-like cells or chondrocyte-like cells [5]. In addition, the risk of VC [6] and osteoporosis [7] increased in patients with CKD, and VC was often accompanied by decreased bone mineral density and bone turnover disorders. The contradictory relationship between bone demineralization and vascular mineralization is often referred to as the “calcification paradox” or “bone-vascular axis” [8, 9]. Both renal osteopathy and VC are crucial components of chronic kidney disease mineral and bone disorder (CKD-MBD) [10]. The “bone-vascular axis” suggests that there may be a close relationship between them. To date, there are few studies on the relationship between VC and renal osteopathy, and systematic studies that combine imaging of VC with bone metabolism markers are even rarer.

This study aims to explore the relationship between CAC and serum bone metabolism markers in MHD patients, analyze the influencing factors for CAC, and attempt to select reliable markers linking vascular calcification and bone metabolism to provide more evidence for the clinical diagnosis and treatment of CKD-MBD.

## Materials and methods

### Subjects

A total of 123 MHD patients in the Department of Nephrology of the Affiliated Hospital of Southwest Medical University from January 2020 to December 2020 were enrolled. There were 79 males (64.23%) and 44 females (35.77%), aged from 22 to 80 years, with a mean age of 56.19 ± 13.18 years. The primary causes of CKD were 45 patients with chronic glomerulonephritis syndrome (36.59%), 29 patients with diabetic nephropathy (23.57%), 24 patients with hypertensive nephropathy (19.51%), 7 patients with polycystic kidney disease (5.69%), and 6 patients with nephrotic syndrome (4.88%), 3 patients with gouty nephropathy (2.44%), 3 patients with urinary tuberculosis (2.44%), and 6 patients with others (4.88%).

Vascular access for all subjects was autologous endovenous fistula or long-term internal jugular vein cuff catheters. They received regular hemodialysis treatments 2 to 3 times per week for approximately 4 h each time. In most subjects, the dialysate contained 1.5 mmol/L calcium ions, but occasionally dialysate containing 1.25 mmol/L or 1.75 mmol/L calcium ions was used depending on the actual condition of the subject. We assessed the dialysis adequacy of the subjects by Ktv values, of which up to 84% had Ktv values of 1.2 or even more, 13% had Ktv values between 1.0 and 1.19, and 3% had Ktv values between 0.60 and 0.99. In addition, these patients received the following medications, including vitamin D and its analogs (Calcitriol, Alfacalcidol, Paricalcitol); Calcium Carbonate and Vitamin D3 Tablet and calcimimetic agent (cinacalcet); phosphorus-binding agents (Sevelamer and lanthanum carbonate), and antioxidant medications (Salmon calcitonin and Risedronate sodium tablets).

The study was approved by the patient’s informed consent and the Clinical Trial Ethics Committee of the Affiliated Hospital of Southwest Medical University (ETHICS Acceptance Number: KY2020007).

### Enrollment criteria

Inclusion criteria: ① age > 18 years; ② dialysis duration ≥ 3 months and dialysis frequency 2 ~ 3 times/week; ③no previous history of peritoneal dialysis, parathyroidectomy, or renal transplantation; and ④ case information was complete and patient able to cooperate. Exclusion criteria: ① estimated survival period < 1 year; ② pregnant, lactating women, or women of childbearing age who plan to become pregnant within the next 6 months; ③ a history of severe infection and surgery in the past 6 months and use of immunosuppressants and glucocorticoids; ④ patients with rickets and primary parathyroid diseases affecting calcium and phosphorus metabolism; ⑤ patients with confirmed or suspected malignant tumors, primary bone tumors or tumor bone metastases affecting bone metabolism; or ⑥ patients with severe malnutrition with albumin < 30 g/L.

### Test grouping

Patients were divided into a calcification group and a noncalcification group. The calcification group was further divided into a mild calcification group (0 < CACS < 400) and a moderate to severe calcification group (CACS ≥ 400) according to the CAC score (CACS).

### Test methods

#### General data collection

The sex, age, height, weight, body mass index (BMI), hemodialysis duration, primary causes of hemodialysis, history of smoking, drinking, hypertension, diabetes mellitus, hyperlipidemia, and osteoporosis were collected and recorded. BMI was calculated as follows: BMI (kg/m^2^) = weight (kg)/height^2^(m^2^).

#### VC assessment and calcification score

The CAC was assessed by scanning the chest with multislice spiral computed tomography (MSCT). Plaques with CT values ≥ 130 HU and areas ≥ 1 mm^2^ were defined as having CAC. Similarly, calcification of the thoracic aorta was defined as the presence of at least one detectable lesion of calcified deposit within the area of the aortic arch and ascending and descending thoracic aorta wall. Heart valve calcification was defined as the presence of at least one detectable lesion of calcified deposit within the area of the aortic and mitral and tricuspid valves. GE Smart Score 4.0 calcification scoring software was used to calculate the CACS by the Agaston scoring method [11].

#### Laboratory parameters

Venous blood samples were collected before dialysis after fasting for at least 8 h. Routine biochemical parameters, including alkaline phosphatase (ALP), albumin (Alb), triglycerides (TG), total cholesterol (TC), high-density lipoprotein cholesterol (HDL-C), low-density lipoprotein cholesterol (LDL-C), glucose (Glu), serum calcium (Ca), serum phosphate (P), and serum magnesium (Mg), were measured by a BS-2000 M automatic biochemical analyzer (Shenzhen Mindray Company. China). When ALB was less than 40 g/L, corrected Ca was calculated by using the formula as follows: corrected Ca (mmol/L) = blood Ca (mmol/L)+[40-Alb (g/L)×0.02]. The product of calcium and phosphorus (Ca×P) was calculated by using the following formula: Ca×P (mg^2^/dl^2^) = corrected Ca (mmol/L) × serum P (mmol/L) × 4 × 3.1. Chemiluminescence immunoassay was used to evaluate calcitonin (CT) (MAGLUMI X8 automatic chemiluminescence immunoassay analyzer, Shenzhen New Industry Biomedical Engineering Co., LTD, China) and total 25-hydroxy vitamin D (25-(OH)D) (Liaision automatic chemiluminescence immunoassay instrument, DiaSorin, Italy); intact parathyroid hormone (iPTH), total type I procollagen amino-terminal peptide (tPINP), N-terminal mid-fragment of osteocalcin (N-MID OC) and β-Type I collagen crosslinked carboxyl-terminal peptide (β-CTX) were measured by automatic electrochemiluminescence immunoassay (cobase-602 automatic electrochemiluminescence immaterialize, Roche, Germany).

### Statistical analysis

The normality of the distribution of all analytes was determined with the Shapiro–Wilk test. Normally distributed continuous data are expressed as the mean ± standard deviation (x ± s), and two independent sample t-tests (Student’s t-tests) were used for comparisons between the two groups. Skew-distributed continuous data are expressed as the median (interquartile interval), and the Mann–Whitney U test was used for comparisons between the two groups. Categorical data are expressed as the frequency (rate), and the chi-square test or Fisher’s exact test was used for comparisons between groups. Pearson correlations were used to determine the correlation between continuous data of normal distribution. Spearman rank correlations were used to determine the correlation between continuous data with a skewed distribution and the correlation between continuous and categorical variables. Univariate linear regression was used to analyze whether N-MID OC was the influencing factor for blood Glu and blood lipids. Influencing factors for CAC and its severity were analyzed by binary logistic regression and adjusted for known or significant confounding factors. A value of *P* less than 0.05 was considered statistically significant. All data analyses were performed using SPSS 25.0 software.

## Results

### Cardiovascular calcification in MHD patients

**Fig. 1:**
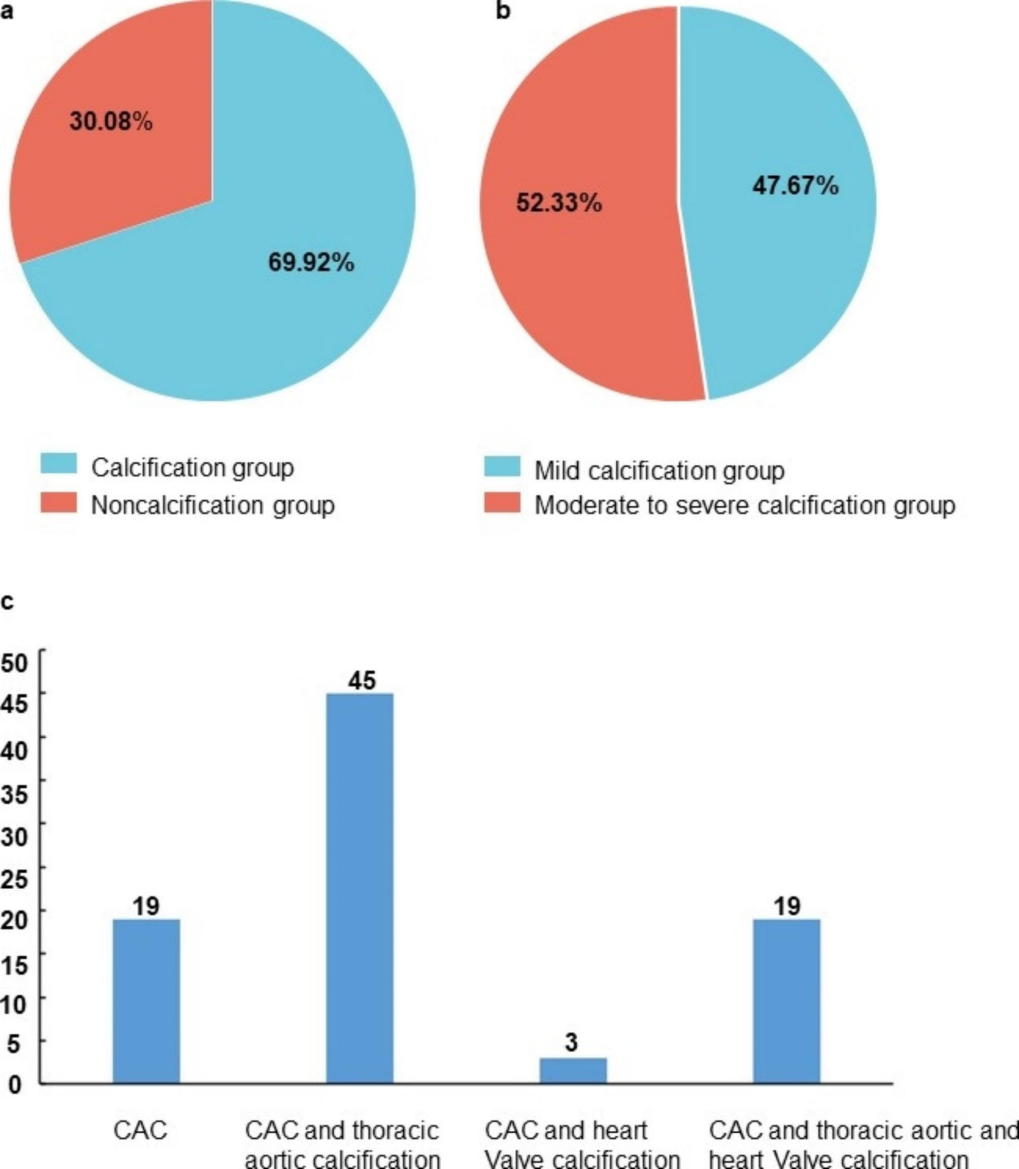
The distribution of vascular calcification. (**a**) Proportion of 123 subjects with and without vascular calcification. (**b**) Proportion of mild and moderate to severe calcification in 86 subjects who developed vascular calcification. (**c**) Detailed information on the site of calcification in 86 subjects who developed vascular calcification

**Vascular calcification is shown in** Fig. 1. A total of 123 MHD patients were enrolled, including 86 patients (69.92%) with CAC and 37 patients (30.08%) without CAC (Fig. 1a). In the calcification group, there were 41 patients (47.67%) with mild calcification and 45 patients (52.33%) with moderate to severe calcification (Fig. 1b), and CACS were(239.22, 230.14 ) and (620.11, 562.58), respectively. In addition, single-site calcification, double-site calcification, and triple-site calcification were further analyzed (Fig. 1c). The proportion of patients in the vascular calcification group with only single-site calcification was 22.09% for CAC, 52.33% for CAC and thoracic aortic calcification, and 3.49% for CAC and heart valve calcification. Of all the patients, 22.09% had calcification at all three sites.

### Comparison of general clinical data and laboratory examination indicators

Compared with the mild calcification group, the moderate to severe calcification group had higher age, BMI, and prevalence of hypertension and diabetes mellitus, whereas there were no statistically significant differences in any of the general clinical data (*P* > 0.05) (Table 1). Serum P and Ca×P levels were significantly higher in patients with moderate to severe calcification when compared to those with mild calcification. Other laboratory examination indicators, such as TG and TC, were slightly higher than those in the mild calcification group; however, this did not reach statistical significance (Table 1). Compared with the noncalcification group, the calcification group had higher age, BMI, and prevalence of hypertension and diabetes mellitus, whereas there were no significant differences in sex, dialysis duration, or other general clinical data (*P* > 0.05) (Table 2. In addition, TC, Glu, P, and Ca×P levels were higher in the calcification group, whereas the Mg concentration was lower; the values of *P* were all < 0.05. However, there was no significant difference in other laboratory examination indicators between the two groups (*P* > 0.05) (Table 2).

**Table 1:** Comparison of general clinical data between the mild calcification and moderate to severe calcification groups in MHD patients

Variables	Mild-calcification group (n = 41)	Moderate to severe calcification group (n = 45)	*t/Z/X* ^2^	*P*
Gender			0.348	0.055
Male, n (%)	28(68.29)	28(62.22%)		
Female, n(%)	13(31.71)	17(37.78)		
Age (year)	58.61 ± 12.87	60.56 ± 10.05	-0.785	0.435
BMI (kg/m^2^)	22.24 ± 3.26	22.89 ± 2.90	0.523	0.603
Hemodialysis duration (months)	38(38)	33(49.5)	-1.475	0.140
Smoking, n(%)	24(58.54)	18(40.00)	2.950	0.086
Drinking, n(%)	21(51.22)	15(33.33))	2.820	0.093
Hypertension, n(%)	39(95.12)	45(100.00)	2.247	0.224
Diabetes mellitus, n(%)	15(36.56)	25(55.56)	3.103	0.078
Hyperlipidemia, n(%)	10(24.39)	18(33.33)	2.381	0.123
Osteoporosis, n(%)	6(14.63)	15(17.78)	0.156	0.693
Alb (g/L)	39.53 ± 2.57	38.98 ± 3.02	-0.908	0.366
TG (mmol/L)	1.52(1.95)	1.65(1.93)	-0.238	0.812
TC (mmol/L)	3.70 ± 1.15	3.97 ± 0.76	-1.260	0.212
HDL-C (mmol/L)	0.95(0.36)	1.02(0.54)	-0.973	0.331
LDL-C (mmol/L)	1.73(1.3)	2.28(0.82)	-1.837	0.066
Glu (mmol/L)	7.875.23)	7.99(5.05)	-0.104	0.613
Ca (mmol/L)	2.19 ± 0.19	2.17 ± 0.15	0.645	0.520
P (mmol/L)	1.68 ± 0.45	1.93 ± 0.52	-2.356	0.021
Ca×P (mg2/dL2)	45.61 ± 12.70	51.73 ± 14.36	-2.086	0.040
Mg (mmol/L)	1.02 ± 0.16	1.03 ± 0.17	-0.333	0.740
ALP (U/L)	96.60(59.40)	79.10(33.50)	-2.079	0.038
CT (pg/mL)	8.07(14.30)	8.08(7.89)	-0.043	0.965
25-(OH)D (ng/mL)	12.96(6.95)	13.80(8.31)	-0.597	0.551
iPTH (pg/mL)	483.99(400.28)	287.09(300.02)	-2.750	0.006
tPINP (ng/mL)	343.80(329.30)	237.00(169.40)	-3.349	0.001
N-MID OC (ng/mL)	163.70(69.15)	102.20(102.11)	-3.411	0.001
β-CTX (pg/mL)	2293(1575.5)	1508(1190)	-2.957	0.003

Abbreviation: BMI body mass index, Alb albumin, TG triglycerides, TC total cholesterol, HDL-C high-density lipoprotein cholesterol, LDL-C low-density lipoprotein cholesterol, Glu glucose, Ca calcium, P phosphate, Ca×P the product of calcium and phosphorus, Mg magnesium, ALP alkaline phosphatase, CT calcitonin, 25-(OH)D 25-hydroxy vitamin D, iPTH intact parathyroid hormone, tPINP total type I procollagen amino-terminal peptide, N-MID OC N-terminal mid-fragment of osteocalcin, β-CTX β-Type I collagen crosslinked carboxyl-terminal peptide

**Table 2:** Comparison of general clinical data between the noncalcification and calcification groups in MHD patients

Variables	Noncalcification group (n = 37)	Calcification group (n = 86)	*t/Z/X* ^2^	*P*
Gender			0.098	0.754
Male, n (%)	23(62.16)	56(65.12)	-	-
Female, n(%)	14(37.84)	30(34.88)	-	-
Age (year)	48.19 ± 13.59	59.63 ± 11.45	-4.796	<0.001
BMI (kg/m^2^)	21.44 ± 3.23	23.06 ± 3.06	-2.644	0.009
Hemodialysis duration (months)	35(38)	35(39)	-0.306	0.759
Smoking, n(%)	17(45.95)	42(48.84)	0.087	0.768
Drinking, n(%)	16(43.24)	36(41.86)	0.020	0.887
Hypertension, n(%)	33(89.19)	84(97.67)	4.014	0.045
Diabetes mellitus, n(%)	5(13.51)	40(46.51)	12.142	<0.001
Hyperlipidemia, n(%)	13(35.14)	27(31.40)	0.165	0.685
Osteoporosis, n(%)	4(10.81)	15(17.44)	0.827	0.363
Alb (g/L)	39.75 ± 2.49	39.24 ± 2.81	0.956	0.341
TG (mmol/L)	1.62(0.91)	1.59(1.88)	-0.709	0.479
TC (mmol/L)	3.47 ± 0.73	3.84 ± 0.97	-2.332	0.022
HDL-C (mmol/L)	1.07(0.41)	0.96(0.45)	-0.474	0.635
LDL-C (mmol/L)	1.91 ± 0.58	2.09 ± 0.83	-1.001	0.317
Glu (mmol/L)	6.4(2.32)	7.94(5.09)	-3.127	0.002
Ca (mmol/L)	2.14 ± 0.21	2.18 ± 0.8	-1.083	0.281
P (mmol/L)	1.53 ± 0.50	1.81 ± 0.50	-2.879	0.005
Ca×P (mg^2^/dl^2^)	40.30 ± 13.39	48.81 ± 13.86	-3.155	0.002
Mg (mmol/L)	1.12 ± 0.19	1.03 ± 0.16	2.561	0.012
ALP (U/L)	99.10(60.90)	87.70(42.38)	-1.685	0.092
CT (pg/mL)	7.51(23.23)	8.08(10.06)	-0.345	0.730
25-(OH)D (ng/mL)	12.87(8.25)	13.12(7.16)	-1.376	0.169
iPTH (pg/mL)	565.01(484.26)	366.27(383.09)	2.165	0.032
tPINP (ng/mL)	450.00(469.20)	281.85(269.08)	-2.765	0.006
N-MID OC (ng/mL)	190.40(82.05)	142.45(118.08)	-2.523	0.012
β-CTX (pg/mL)	2672(2394)	1812(1462)	-2.570	0.010

Abbreviation: BMI body mass index, Alb albumin, TG triglycerides, TC total cholesterol, HDL-C high-density lipoprotein cholesterol, LDL-C low-density lipoprotein cholesterol, Glu glucose, Ca calcium, P phosphate, Ca×P the product of calcium and phosphorus, Mg magnesium, ALP alkaline phosphatase, CT calcitonin, 25-(OH)D 25-hydroxy vitamin D, iPTH intact parathyroid hormone, tPINP total type I procollagen amino-terminal peptide, N-MID OC N-terminal mid-fragment of osteocalcin, β-CTX β-Type I collagen crosslinked carboxyl-terminal peptide

### Comparison of serum bone metabolic markers

Compared with the mild calcification group, the levels of ALP, iPTH, tPINP, N-MID OC and β-CTX were significantly lower in the moderate to severe calcification group (*P* < 0.05), whereas CT and 25-(OH)D levels showed no significant differences between the two groups (*P* > 0.05) (Table 1). Compared with the noncalcification group, iPTH, tPINP, N-MID OC, and β-CTX levels were significantly lower in the calcification group (*P* < 0.05), whereas there were no statistically significant differences in ALP, CT, and 25-(OH)D between the two groups (*P* > 0.05) (Table 2), which indicates that the degree of bone turnover in the calcification group is lower than that in the noncalcification group. Compared with the mild calcification group, the degree of bone turnover in the moderate to severe calcification group was more inactive.

### Correlation between CACS and all parameters

Spearman rank correlation analysis showed that the CACS was positively correlated with TC (r = 0.253, P = 0.019), LDL-C (r = 0.227, P = 0.036), P (r = 0.213, P = 0.049), and Ca × P (r = 0.232, P = 0.032) and negatively correlated with N-MID OC (r = -0.214, P = 0.047) and β-CTX (r = -0.223, P = 0.039). There was no statistically significant correlation between CACS and ALP, CT, 25-(OH)D, iPTH, tPINP, general clinical data, or other laboratory parameters.

### Influencing factors for CAC and the severity of CAC

A binary logistic regression model was established to analyze the influencing factors for the severity of CAC by taking the occurrence of severe CAC as the dependent variable and the indicators of statistically significant differences between mild and moderate to severe CAC as the independent variables. Univariate regression analysis showed that age, P, and Ca×P were risk factors for severe CAC, and tPINP and N-MID OC were protective factors for severe CAC. Whereas, iPTH and β-CTX had no significant influence on the severity of CAC (Table 3). Following adjustment for confounding factors, including sex, dialysis duration, smoking, drinking, hypertension, hyperlipidemia, and osteoporosis, the risk factors remained consistent. N-MID OC was still an independent protective factor, whereas tPINP was no longer an independent protective factor (Table 3).

**Table 3:** Univariate and multivariate analysis of influencing factors for the severity of CAC in MHD patients

Variables	Univariate analysis					Multivariate analysis					
	B	Wald	P	OR	95%CI of OR	B	Wald	P	OR	95%CI of OR	
P	1.058	5.059	0.025	2.881	1.146–7.242	1.518	6.923	0.009	4.563	1.473–14.139	
Ca×P	1.579	4.022	0.045	1.035	1.001–1.071	0.051	6.099	0.014	1.053	1.011–1.096	
ALP	-0.005	1.802	0.179	0.995	0.988–1.002	-0.002	0.333	0.564	0.998	0.990–1.006	
iPTH	-0.001	2.887	0.089	0.999	0.998-1.000	0.000	0.249	0.618	1.000	0.999–1.001	
tPINP	-0.002	4.527	0.033	0.998	0.997-1.000	-0.001	0.765	0.82	0.999	0.998–1.001	
N-MID OC	-0.012	9.478	0.002	0.988	0.981–0.996	-0.012	6.577	0.010	0.988	0.979–0.997	
β-CTX	0.000	5.617	0.018	1.000	0.999-1.000	0.000	1.169	0.280	1.000	0.999-1.000	

Abbreviation: P phosphate, Ca×P the product of calcium and phosphorus, ALP alkaline phosphatase, iPTH intact parathyroid hormone, tPINP total type I procollagen amino-terminal peptide, N-MID OC N-terminal mid-fragment of osteocalcin, β-CTX β-Type I collagen crosslinked carboxyl-terminal peptide

A binary logistic regression model was established to analyze the influencing factors for CAC by taking the occurrence of CAC as the dependent variable and the indicators of statistically significant differences as the independent variables. Univariate regression analysis showed that age, BMI, TC, Glu, P, and Ca×P were risk factors for CAC, whereas not having diabetes mellitus, Mg, low iPTH, tPINP, and N-MID OC were protective factors for CAC (Table 4). Following adjustment for covariables, including sex, hemodialysis duration, smoking, drinking, hypertension, hyperlipidemia, and osteoporosis, the risk factors remained consistent. Not having diabetes, Mg, and N-MID OC were still independent protective factors for CAC, whereas the relationship between iPTH or tPINP and CAC was lost (Table 4).

**Table 4:** Univariate and multivariate analysis of influencing factors for CAC in MHD patients

Variables	Univariate analysis					Multivariate analysis				
	B	Wald	P	OR	95%CI of OR	B	Wald	P	OR	95%CI of OR
Age	0.071	16.265	<0.001	1.074	1.037–1.112	0.074	15.060	<0.001	1.077	1.037–1.118
BMI	0.176	6.399	0.011	1.192	1.040–1.367	0.209	7.633	0.006	1.232	1.063–1.429
Hypertension	-1.627	3.343	0.067	0.196	0.034–1.124	-1.448	2.339	0.126	0.235	0.037–1.503
Diabetes mellitus	-1.717	10.599	0.001	0.180	0.064–0.505	-1.833	10.220	0.001	0.160	0.052–0.492
TC	0.482	4.126	0.042	1.619	1.017–2.577	0.614	5.537	0.019	1.849	1.108–3.084
GLU	0.156	5.175	0.023	1.169	1.022–1.337	0.154	4.665	0.031	1.166	1.014–1.341
P	1.190	7.433	0.006	3.286	1.397–7.727	1.236	7.336	0.007	3.443	1.407–8.425
Ca×P	0.050	8.703	0.003	1.052	1.017–1.087	0.052	8.414	0.004	1.053	1.017–1.091
Mg	-2.925	5.945	0.015	0.054	0.005–0.563	-3.482	7.302	0.007	0.031	0.002–0.384
iPTH	-0.001	5.009	0.025	0.999	0.999-1.000	-0.001	2.982	0.084	0.999	0.998-1.000
tPINP	-0.001	4.669	0.031	0.999	0.998-1.000	-0.001	2.897	0.089	0.999	0.998-1.000
N-MID OC	-0.008	5.938	0.015	0.992	0.985–0.998	-0.008	5.717	0.017	0.992	0.986–0.999
β-CTX	0.000	5.929	0.015	1.000	0.999-1.000	0.000	4.157	0.041	1.000	0.999-1.000

Abbreviation: BMI body mass index, TC total cholesterol, Glu glucose, P phosphate, Ca×P the product of calcium and phosphorus, Mg magnesium, iPTH intact parathyroid hormone, tPINP total type I procollagen amino-terminal peptide, N-MID OC N-terminal mid-fragment of osteocalcin, β-CTX β-Type I collagen crosslinked carboxyl-terminal peptide

### Univariate regression analysis for correlates of N-MID OC and blood glu and blood lipids

In all patients, univariate linear regression analysis showed that N-MID OC negatively affected Glu (B= -0.021, P<0.001), TC (B= -0.003, P = 0.012), and LDL-C (B= -0.003, P = 0.013) and did not affect TG (b = -0.003, P = 0.223) or HDL-C (b = -0.000, P = 0.535). In the subgroup of patients with CAC, the univariate linear regression analysis still showed that N-MID OC negatively affected Glu (B= -0.021, P = 0.003), TC (B= -0.004, P = 0.015), and LDL-C (B= -0.003, P = 0.017) and did not affect TG (B= -0.003, P = 0.375) or HDL-C (B= -0.001, P = 0.424).

## Discussion

CKD-MBD is one of the most common complications in patients with CKD, especially in MHD patients, and VC is a crucial component of CKD-MBD, which is closely related to the incidence and mortality of cardiovascular diseases [10]. The CDCS has shown that the CAC is the most common calcification site in dialysis patients in China [4]. In this study, MSCT was used to assess CAC. The results showed that the rate of CAC in MHD patients was 69.92%, which was highly consistent with the 72.1% reported by the CDCS. A study from Italy showed that the CAC rate in MHD patients was 87.7% [12]. Therefore, our results are consistent with those of domestic and foreign studies, which indicates that VC is highly prevalent in MHD patients.

Renal osteodystrophy is defined as abnormal bone histology in CKD-MBD, including abnormal bone conversion, bone mineralization, and bone volume. Renal osteodystrophy and vascular calcification are both important components of CKD-MBD, and previous studies have revealed a possible “bone-vascular axis” crosstalk between them. Our previous studies by modeling vascular calcification in CKD rats showed that the rate of bone turnover was reduced in CKD rats that developed vascular calcification. In addition, a further decrease in bone conversion rate occurred with increasing severity of vascular calcification[13]. Encouragingly, these results are consistent with the current clinical study. Specifically, iPTH is considered to be the most valuable bone metabolic marker for evaluating bone turnover [14, 15], and lower iPTH means a greater possibility of low bone turnover. tPINP is an additional amino-terminal peptide released by type I procollagen in the process of osteoblast synthesis of osteocollagen, and its concentration in serum can reflect the activity of osteoblasts. In the process of bone resorption, β-CTX, the degradation product of type I collagen, can reflect the bone resorption activity of osteoclasts. N-MID OC is mainly involved in the differentiation and proliferation of osteoblasts, maturation of the extracellular matrix, and mineralization of the bone matrix, as well as is generally considered a marker of bone formation. Furthermore, N-MID OC deposited in the bone matrix can also be released into the blood circulation during bone resorption. Thus, N-MID OC can reflect the overall level of bone turnover. The above results indicated that the bone turnover activity in the calcification group was lower than that in the noncalcification group. With the aggravation of VC, bone turnover became more inactive. Our results were consistent with those of London et al., who found that VC in patients with end-stage renal disease (ESRD) was associated with low bone turnover confirmed by bone histopathology [16]. In addition, Barreto et al. also showed that low bone turnover in hemodialysis patients is associated with the progression of the CACS [17]. We speculate that, under relatively low bone turnover, circulating Ca and P cannot be effectively deposited on the surfaces of bones, and the ability of bones to buffer circulating Ca and P decreases, resulting in high levels of circulating Ca and P. The results of the present study also show that higher serum P and Ca×P were independent risk factors for CAC and were positively correlated with the severity of CAC. Previous literature has also shown that high serum P levels can promote the occurrence and development of VC by inducing VSMCs to transdifferentiate into osteoblast-like cells or chondrocyte-like cells and promoting the expression of the osteogenic transcription factor Msx2 or cartilage formation transcription factor Sox9 [18]. The increased Ca×P can also directly promote extraosseous ectopic mineralization and exaggerate VC. Phenotypic osteoblasts and osteocytes in calcified vessels can secrete sclerostin (SOST), Dickkopf-related protein 1 (DKK1), etc. These proteins can inhibit VC by inhibiting Wnt/β-catenin signaling. In addition, it may also inhibit the Wnt/β-catenin signaling of osteoblasts in bone and thereby inhibit bone formation, which causes a decrease in bone turnover and an increase in serum Ca and P followed by a vicious cycle and exacerbates VC [19–21]. Therefore, strictly controlling disorders of Ca and P metabolism is still an important measure to prevent the occurrence and development of VC.

In the present study, we demonstrated that reduced serum OC promotes coronary artery calcification. In a previous model of vascular calcification in CKD rats, we also showed that reduced serum OC correlated with the severity of vascular calcification[13]. Approximately 36% of the studies conducted in Asia reported a negative correlation between serum OC concentrations and VC or arteriosclerosis [22]. In hemodialysis patients, Botond Csiky [23] found that there was a negative correlation between serum OC and carotid-femoral pulse wave velocity, an alternative index of VC, and lower serum OC levels increased the risk of cardiovascular disease [24]. However, the current findings are still controversial. N-MID OC has not been considered a protective or risk factor for VC. A basic study has shown that OC can induce VSMC calcification by decreasing glycolysis and maximal respiration of VSMCs [25]. The results of Reyes Garcia [26] showed that the serum OC concentration of male patients with coronary heart disease was significantly higher than that of patients without coronary heart disease, and the serum OC level of female patients with aortic calcification, carotid intima-media thickness thickening or carotid plaque was also significantly higher. Other studies have also found that OC is positively correlated with CAC in patients with acute myocardial infarction, and OC, as a regulator of mineralization, is considered a promoting factor for VC [27]. However, we are more inclined to suggest that serum N-MID OC is a protective factor for VC, which is speculated to contribute to the balance of glucose and lipid metabolism [28, 29] by regulating the expression of insulin genes, β-cell proliferation, and the expression and release of adiponectin in adipocytes. Further studies are needed to reveal the anti-calcification mechanisms of N-MID OC in VC. N-MID OC can not only reflect the situation of bone metabolism but also contribute to evaluating the occurrence and severity of VC. Therefore, N-MID OC may be a reliable serum bone metabolism marker linking VC and bone metabolism in MHD patients.

iPTH is an important factor in regulating Ca and P metabolism. This study’s univariate analysis showed that low serum iPTH contributed to CAC, but the conclusion was invalid in multivariate analysis. Similar to iPTH, the bone formation marker tPINP and bone resorption marker β-CTX could not independently affect CAC. Therefore, our study indicates that the main significance of iPTH, tPINP, and β-CTX in clinical practice is to indirectly assess the state of bone turnover, and the relationship between iPTH, tPINP, β-CTX, and VC should be analyzed from the insight of abnormal bone turnover. Whether the concentrations of serum iPTH, tPINP, and β-CTX in MHD patients are directly related to VC needs further investigation.

Our study also showed that hypomagnesemia was an independent risk factor for CAC. Montezano et al. [30] found that adding Mg ions to high-P media can prevent osteogenic transdifferentiation and calcification of VSMCs and promote the expression of anti-calcification proteins, including osteopontin and matrix GLP proteins. Many clinical studies have shown that serum Mg is negatively correlated with VC. Hypomagnesemia is an independent risk factor for VC and is closely related to the risk of cardiovascular disease and mortality in hemodialysis patients [31, 32]. Some studies have shown that Mg ions can inhibit the transdifferentiation of VSMCs into osteoblast-like cells and the formation of hydroxyapatite crystals [33], but the specific mechanism by which Mg ions regulate VC is still unclear. Moreover, we found that traditional factors, such as old age, high BMI, high cholesterol, and diabetes mellitus, were still risk factors for CAC, suggesting that strict control of body weight, blood sugar, and blood lipids, as well as strengthening the management of chronic diabetes mellitus, are effective measures to reduce the incidence of VC in MHD patients.

There are several limitations to our study. First, it is a single-center study with a relatively small sample size. Second, our study is a cross-sectional study, which only discusses the relationship between bone metabolic markers and vascular calcification, but the causal relationship cannot be determined, especially in the analysis of the prognosis or end events of patients.

## Conclusions

In conclusion, our study indicated that there is a high incidence of CAC in MHD patients. CAC is closely related to abnormal bone metabolism and markers of bone metabolism. Relatively low bone turnover can promote the occurrence and development of CAC. Decreased N-MID OC is an independent risk factor for CAC and the severity of calcification in MHD patients. N-MID OC may be a reliable serum bone metabolism marker linking VC and bone metabolism, and it is expected to become a new target for the treatment of CKD-MBD, which needs to be further investigated in the future.

## Data Availability

All authors make sure that all data and materials support their published claims and comply with field standards. The datasets used and analyzed during the current study are available from the corresponding author upon reasonable request. The data supporting this study’s findings are available from the corresponding author, Santao Ou, upon reasonable request.
